# Report on Meteorology

**Published:** 1859-06

**Authors:** 


### REPORT ON METEOROLOGY,

We notice that J. G. Westmoreland, M. D., of Atlanta, Ga., was appointed at the late meeting of the American Medical Association, to make a Report to the next meeting upon Meteorology, a subject in which he has for a length of time been much interested, and in taking observations in regard to which, for the Smithsonian Institute, he is now engaged.

